# Letter from Professor Martins

**Published:** 1840-04-18

**Authors:** 


### MEDICAL EXAMINER.

DEVOTED TO MEDICINE, SURGERY, AND THE COLLATERAL SCIENCES.

No. 16.] PHILADELPHIA, SATURDAY, APRIL 18, 1840. [Vol. III.

### FOREIGN CORRESPONDENCE.

### LETTER FROM PROFESSOR MARTINS.

No. IV—Concluded.

*Spirit of the French Medical Journals, and their influence during the year* 1839.

Paris, 21st February, 1840.

To the Editors of the Medical Examiner.

The indifference felt in France for every thing beyond her limits, has long been to her a subject of reproach with strangers. Among the causes of this apparent indifference, has been principally the system of centralization established under the republic, which made the state the most compact, the most of a unit in Europe. Paris, the immense centre of this great whole, has within itself so many resources, so many lights, that our French savans, residing almost all in the capital, finally persuaded themselves that there was nothing of consequence beyond its walls. The admiration of strangers, who, whether from a sense of justice or of politeness, complimented us on the number and grandeur of our institutions, served only to aliment these feelings of pride. Napoleon, with all his power and influence, cherished it. Like all despots, he wished to isolate his own from every other people, whom he constantly represented to us as destined one day to become tributary to the French empire. Napoleon fell; foreign armies twice occupied France, and the feelings of hatred towards foreigners was but deepened. We then became exlusive from patriotism. Gradually, however, our national antipathies were weakened—it began to be understood that nations gained nothing in these wars waged for the advantage of kings; and we came to cast eyes of curiosity first upon England, then upon Germany and America. This taste for erudition took its birth at the epoch of the revolution of July, which awakened the sympathies of all enlightened nations towards France. Philology gave the impulse, and medicine soon received it.

In books, in the *concours* of the School of Medicine, in clinical lectures, in the very theses, foreign authors began to be cited, the periodicals of Germany, of England, and of America were consulted, and we were astonished at the rich stores they contained. Two individuals chiefly contributed to bring about this result, MM. Littré and Dezeimeris. To extend the study of books, as well as of facts, they founded, in 1837, the journal entitled *L'Expérience.* M. Littré having since devoted his whole time to the publication of the works of Hippocrates, text and translations, M. Dezeimeris has taken the sole editorial charge of the journal. I shall cite his remarks upon entering on the duty. "It is my intention," said he, "still to devote myself to the task of comparing and weighing every new fact and doctrine to which public attention is accidentally called, with analogous facts, to be found in the works of all countries and times, which may aid in the solution of the question in controversy.

"In the domain of experimental study, there is no science or art which can be advanced merely by adding isolated facts to facts already in existence: this is to increase the materials for future *science*, but not to promote actual science. Medicine demands imperiously, whether we consider it merely as the healing art or as a science, that, out of the frightful mass of observations which have been unceasingly accumulating, should emerge the practical results to which they lead, and the general principles which induction can extract from them. We shall, therefore, devote ourselves less to the publication of new and isolated cases, than to place side by side with those on which public attention dwells, facts of analogous nature which have been forgotten; that by comparison we may arrive at conclusions."

M. Dezeimeris has kept his word. Librarian of the faculty, and in possession of an immense collection of foreign reviews and books, he has devoted his erudition to all the great questions which have agitated the Academy. If his name does not always figure in these debates, it is because he has placed his knowledge at the disposal of the disputants, who quote the authors pointed out to them by him, without quoting himself. The following are the principal memoirs published in L'Expérience during 1839 :

On the pathological anatomy of old dislocations of the femur, by M. Sédillot. On the treatment of dysentery with albumen, given in drinks and by injections, by M. Mondierè. On albimunuria, by M. Martin Solon. On the blood of scrofulous subjects, by M. Dubois, of Amiens. Researches on ruptures of the heart, by M. Dezeimeris. On spontaneous rupture of the womb at different periods of pregnancy, by the same writer. Clinical researches on the coagula formed during life in the blood vessels, by M. Bouillaud. On rheumatism of the uterus during pregnancy and confinement, by M. Dezeimeris. On diseases of the frontal sinuses, by the same writer. On the anatomical phenomena produced by the development of tuberculous mattter, around the bony articulations, by M. Guillot. On diseases of the epiglottis, by M. Dezeimeris.

I come next to a class of journals very difficult to characterize, no general idea or practical doctrine having presided at their creation. Not that I cast this up to them as a reproach, it is merely a difficulty that I point out: for if it was easy to indicate the tendency and spirit of the reviews of which I have before spoken, it is really impossible to do so in the case of those which we are about to speak of. I shall confine myself, then, to an estimate of their influence and utility.

The *Medical Gazette* is the most widely circulated of the French medical journals. It is that which pleases most, not only our native, but also foreign physicians. This journal appears weekly and keeps the reader *au courant* of whatever takes place of importance throughout the entire world of medicine. Its first pages are filled with original memoirs, in general, short and not drawn out, but treating of subjects the most varied. Next comes an analysis of the French or foreign journals, of the sittings of the academy, scientific news, &c. Through its means, the reader is enabled to follow the concours of the Faculty, and estimate the chances of the competitors. Lastly, he is presented with a page devoted to the news, *on dits*, and gossip, connected with medical topics. No one is named, but every body is spoken of, so as to be recognised without being mentioned. To the physician of Paris, especially, who is familiar with all the personages alluded to, this is a particular treat, of the kind that we French so much enjoy—scandal told with wit and point. The Gazette, however, often takes up graver subjects of discussion, connected with the profession, with the positions of physicians and apothecaries, and their relations to the actual condition of society. It dwells upon the fact, that our social position in France is not what it should be, and that if physicians are the class of citizens from whom the widest range of service is demanded, they are far from participating most largely in the favours of power. Hence, the Medical Gazette exercises great influence as often as the material interests of physicians are brought into question. It is the field upon which take place all the contests on the topics of mecical responsibility and medical organization. It insists, with laudable perseverance, upon a new organization of medicine, which shall put an end to the progress of charlatanism ; it demands the suppression of health officers, a limitation of the number of apothecaries, and more rigid examinations for the Doctorate ; in a word, it stands up for all those reforms which the French physicians have been so long looking for. The principal original papers published in 1839, in this journal, are : Upon organic strictures of the rectum, by MM. Berard and Maslieurat. Exposition of researches made upon some parts of the brain, by M. Hives. On mammary abscesses of females, by M. G. Jeanselme. On luxation of the superior extremity of the ulna, by M. Sédillot. On the cure of strictures of the urethra, by M. Heybard. On fractures of the femur and the neck of the humerus, by M. Bonnet. Account of an epidemic of the sweating disease at Coulommiènes, by MM. Barthez, Gueneau de Mussy, and Landouzy.

The *Journal des Connaissances Medico-Chirurgicales* was established in 1832, by MM. Trousseau, Gouraud, and Lebaudy. It appears monthly, and gives a tolerably satisfactory analysis of the more important productions in medicine. Its principal merit is the publication of very good anatomical plates, intended to recall to distant physicians the situation of those important parts which it behooves both surgeon and physician constantly to have fresh in his remembrance. Hence, this journal is more particularly intended for the country practitioner. Some interesting papers appeared in this journal in 1839. On blisters, by M. Trousseau. On facial hemiplegia neonatorum, by M. Landouzy. On typhoid fever, by M. Taupin. On puerperal fever, by M. Voillemier. On serous apoplexy, by M. Boudet. On gangrenous stomatitis, by M. Taupin.

The *Journal des Connuissances Medicales et de Pharmacologie*, edited by an association of physicians and apothecaries, disclaims all scientific pretensions; its only pretensions are to be useful. Its sole end is to keep practitioners au courant of all discoveries susceptible of real and immediate application to the healing art. Therapeutics is, therefore, its principal object, but not its only one. There is in France, actually, no journal of pharmacy. That which bears the title contains, in fact, only analytical memoirs of high chemistry, but does not teach the apothecary the new processes, the manipulations to be preferred in the preparation of remedies. The journal of which we speak, under the able direction of the pharmaceutic editor, M. Vée, presents to the apothecary whatever it is important he should know for the interest of public health, while, in the same manner, in the medical department of the journal, theory is always sacrificed to practice.

The list of memoirs published in 1839, sufficiently attests the character of the Journal. On the biniodide of mercury, and the iodohydrargyrate of the iodide of potassium, in the cure of syphilis, by M. Pache. On the radical cure of circocele, by M. Roux. On the accidents occasioned by gas used for lighting, by M. Marc Moreau. Intermittent meningitis cured by sulphate of quinine, by M. V. Jannizot. On diabetes, by M. Bouchardat.

The *Bulletin de Therapeutique*, by M. Miguel, was, for a long time, successful. It was well edited, and contained therapeutic articles, short, but good. Every physician seemed to consider this journal as an arsenal, containing an inexhaustible store of arms, to combat disease. But it soon began to be discovered that these remedies, so potent on paper, often failed at the bedside, and the Bulletin de Theraputique began to see, and continues to see a falling off in its list of subscribers.

What shall I say of the *Gazette des Hopitaux*, or *French Lancet?* The title is that of a journal of medicine. It is even, generally, headed by two or three cases of disease—but what cases! incomplete, always inexact, and miserably put together. It has always struck me, that these cases were merely a pretence to give the Journal the title of a journal of medicine; its principal object appearing to be the publication of violent diatribes against its enemies, which it contains daily, and the sheet of advertisements.

Two new journals have recently appeared—the *Esculapius*, and the *Journal of Practical Physicians;* but, their existence has been too brief to allow any estimate to be formed of their ability, and probable value. I doubt, however, whether they are destined to play an important part, for, represented as every medical doctrine and interest appears to be just now, new comers are unlikely to find a place.

